# Treatment of Malarial Fever

**Published:** 1890-01-11

**Authors:** 


### TREATMENT OF MALARIAL FEVER.

The fact that quinine does occasionally prove unavailing in the treatment of malarial fevers has suggested experiments with other drugs as arsenic, and the so-called Warburg's tincture has enjoyed a considerable reputation in India, though it is, after all, but an aromatic compound of bark or quinine with aloes. But in 1877 a Mr. Clark made in the *Lancet* the astounding assertion that having treated 5,000 cases with picrate of ammonium, he had only failed to effect a cure in nine, eight of which derived no benefit from quinine. On the strength of this statement Prof. Liebreich suggested to the German New Guinea Company a trial of the new remedy, and Dr. Oscar Schellong, then in their service, made a number of careful observations alike on Europeans, Malays, and Melanesians, using, however, larger doses than Clark. He was thoroughly disappointed with the results in all his cases; in a few, indeed, it seemed to exert a slight influence, but these were of natives of the country, in whom, from long "saturation," the attacks, even when untreated, are generally milder than those experienced by newly-arrived Europeans. So far as he could see, it neither averted relapses nor moderated their severity, while it certainly had no effect in reducing the enlargement of the spleen, the only result which, as Dr. Shellong properly observes, could be described as a cure. The term is not applicable to the termination of an attack, which tends, even without treatment, to cease spontaneously in almost every instance. Some weeks ago we called attention to the advantage stated by Italian physicians to attend the combination of ergotin with quinine in avoiding cinchonism. Digitalis is said to aid the characteristic action of the drug in malaria; but Dr. Tibiriçà, while not questioning its value, maintains that pereirine is greatly superior; ten grains each of quinine and of pereirine being at least equal to twenty of quinine, without the ill effects that may follow so large a dose. Even when quinine fails to control the fever in the least, however large and well borne the doses, the addition of pereirine is often followed by the happiest results, and no less so when the quinine alone had only increased the depression caused by the fever it failed to reduce.

